# Sap Flow Variability in *Malus domestica* Borkh. (‘Jazz^TM^’) Trees Under Differing Water Supply Conditions and Fruit Loads

**DOI:** 10.3390/plants15040608

**Published:** 2026-02-14

**Authors:** Evangelos Xylogiannis, Mohammad Yaghoubi Khanghahi, Rosangela Addesso, Alejandro Galindo, Bartolomeo Dichio, Brent Clothier, Steve Green, Adriano Sofo

**Affiliations:** 1 RS Fruit SA, 2nd km Monospita–Chariessa, 59035 Naoussa, Greece; evangelos.xylogiannis@gmail.com; 2 Department of Agricultural, Forestry, Food and Environmental Sciences (DAFE), Università degli Studi della Basilicata, Via dell’Ateneo Lucano 10, 85100 Potenza, Italy; rosangela.addesso@unibas.it (R.A.); bartolomeo.dichio@unibas.it (B.D.); adriano.sofo@unibas.it (A.S.); 3 Department of Irrigation, CEBAS-CSIC, University of Murcia, P.O. Box 164, E-30100 Espinardo, Murcia, Spain; agegea@us.es; 4 The New Zealand Institute for Plant and Food Research Limited, Palmerston North 4447, New Zealand; brent.clothier@plantandfood.co.nz (B.C.); steve.green@plantandfood.co.nz (S.G.)

**Keywords:** apple trees, dry matter content, leaf area index, maximum daily shrinkage, sap flow, water management

## Abstract

Efficient apple orchard water management under climate variability requires understanding how fruit load and water supply regulate branch-scale water use to optimize irrigation, yield, and fruit quality. During the summer of 2014, sap flow (SF) and maximum daily shrinkage (MDS) were measured in one branch from six apple trees (Malus domestica Borkh. Cv. ‘Jazz™’) using the Compensation Heat Pulse method and diameter variation sensors in an orchard near Havelock North, New Zealand. One west-oriented branch per tree, with diameters of 1.5 to 2.3 cm, was monitored alongside midday stem (ψs) and leaf (ψl) water potentials, leaf gas exchanges, leaf area index (LAI), and fruit dry matter per branch at the end of the growing season. Half of the trees were subjected to irrigation withdrawal after day of year (DOY) 31 (non-irrigated treatment), resulting in a significantly lower midday stem water potential (ψs) by DOY 56 (−1.03 MPa). Pre-harvest, SF and MDS were tightly correlated (r^2^ = 0.69), but this correlation decreased post-harvest (r^2^ = 0.16) due to reduced fluctuations in both SF and branch variations (BV). SF was normalized per unit of leaf area, categorizing branches into high and low LAI: fruit dry matter ratio. SF values were approximately 2.2 times higher for FI pre-harvest and remained 2-fold higher post-harvest, associated with lower ψl and higher midday leaf transpiration for FI. MDS was identified as a better indicator of mild water deficit compared to SF, with both measurements responding effectively to midday vapor pressure deficit and reference evapotranspiration values. Overall, MDS proved to be a more sensitive indicator of mild water deficit than SF, while fruit load exerted a persistent influence on branch water use, highlighting the value of branch-scale measurements for improving irrigation management in apple orchards.

## 1. Introduction

Understanding the water dynamics in apple trees is critical for optimizing irrigation and enhancing fruit production, especially under varying fruit loads and water supplies. Recent advancements in thermometric techniques have provided robust methodologies for directly measuring sap flow (SF) and assessing the water status of trees [1]. In particular, SF measurements have seen significant improvements over the past few decades, allowing for continuous and precise data acquisition without adversely impacting plant growth and physiology [2].

Apple trees are known to exhibit strong physiological sensitivity to irrigation management, particularly during the fruit growth and pre-harvest period [3,4]. Deficit irrigation has been shown to reduce midday stem water potential, alter stomatal conductance, and modify sap flow dynamics, with consequences for fruit growth, dry matter accumulation, and yield stability [5,6]. In apple orchards, mild water deficits often develop gradually and may not immediately affect gas exchange, making continuous indicators such as sap flow and stem diameter variation especially valuable for irrigation scheduling [7]. However, the combined response of sap flow and maximum daily shrinkage to irrigation withdrawal at the branch scale remains insufficiently documented.

Numerous studies have documented SF dynamics in field-grown apple trees. For instance, research conducted in New Zealand indicated that mature apple trees with a leaf area of approximately 45 m^2^ can consume around 70 L day^−1^ per tree, with peak daily consumption reaching approximately 6 L day^−1^ [8]. In another study, Li et al. [9] reported hourly water fluxes for mature Golden Delicious apple trees to be about 0.30–0.40 L tree^−1^ and 0.40–0.50 L tree^−1^ during their first and second years of measurement, respectively, with an associated leaf area of roughly 15 m^2^. In addition to trunk measurements, SF has also been assessed in tree branches [10]. It has been reported that SF varied significantly among branches in pecan tree (*Carya illinoensis* cv. Wichita) [11], with strong correlations to radiation and light interception [12]. Conversely, Du et al. [13] observed no difference in SF among branches situated on different sides of the tree when applying alternated partial root-zone drying.

Apple trees transpiration and water use are profoundly influenced by climatic conditions [14]. Thus, measuring environmental variables becomes crucial in understanding tree water status, potentially even more so than assessing soil moisture variations. Indicators such as leaf water potential and midday stem water potential provide valuable insights into plant-water relations [15,16]. Multiple studies have shown that maximum daily shrinkage (MDS) and trunk diameter variations (TDV) correlate significantly with these water relation indicators, reflecting the overall water status of the plant [17,18,19]. Moreover, monitoring stem diameter variations, specifically MDS, provides complementary insights into stem behavior in response to environmental conditions. MDS serves as an indicator of physiological and water status in trees, demonstrating relationships with climatic variables such as temperature and evapotranspiration. Consequently, accurate evaluations of plant water stress can enhance water use efficiency through improved and cost-effective water management strategies [20]. In addition to SF measurements, the assessment of stem diameter contraction has gained traction as a means to relate stem micro-fluctuations to water deficits and environmental variables [21].

The compensation heat pulse method (CHPM) has already introduced as a valuable tool in measuring SF that operates with minimal instrumentation and power requirements, making it accessible for various applications in plant physiology and horticulture [22]. Despite its practical advantages, CHPM has limitations in accuracy, particularly for low SF rates. This inaccuracy arises due to the dispersion of heat pulses through conduction before they reach the sensors, leading to erroneous temperature readings [23]. Several studies have attempted to identify the threshold at which CHPM can reliably measure SF, with estimates varying across research. Some preliminary studies have indicated that effective measurement ranges fall between approximately 3.0 and 15.7 cm/h, underscoring the importance of exercising caution when interpreting results at lower flow rates [23,24]. Despite these limitations, CHPM has seen extensive use in fields such as horticulture and physiological assessments, demonstrating its importance in understanding plant water transport mechanisms. The method involves placing temperature sensors at specified distances from a heater in the xylem, allowing for the calculation of heat pulse velocity (HPV) based on temperature changes. However, the installation of probes can damage xylem tissues [25], necessitating corrective measures to account for these wounds when calculating SF. To derive actual SF rates from HPV, it is essential to consider the physical properties of the wood and sap, including moisture content and density [25,26]. This process requires careful sampling at varying depths within the sapwood due to the non-uniformity of SF density. Once SF density is determined, total volumetric SF can be estimated through integration techniques. Nevertheless, while CHPM has specific limitations, it remains a crucial method for effectively studying SF and understanding the physiological responses of plants to their environment.

Based on what was discussed above regarding the water dynamics in trees, the primary objective of this study was to evaluate the response of branch-level SF and MDS in apple trees subjected to contrasting irrigation regimes (irrigated vs. irrigation withdrawal) during the summer period. Specifically, we aimed to (i) assess the sensitivity of SF and MDS to mild water deficit induced by irrigation cessation, (ii) examine pre- and post-harvest changes in the relationship between SF and MDS, and (iii) quantify the influence of fruit load, expressed as the leaf area index to fruit dry matter ratio, on branch water use. We hypothesized that irrigation withdrawal would primarily affect MDS before significantly reducing SF, and that branches with higher fruit demand would exhibit higher SF and stronger coupling with atmospheric demand.

Despite extensive research on SF and stem diameter variations in fruit trees, their combined response at the branch scale under contrasting irrigation regimes and varying fruit loads remains poorly documented. This study provides novel insights by simultaneously quantifying branch-level sap flow and maximum daily shrinkage before and after harvest, thereby elucidating the role of fruit load in modulating tree water use under mild water deficit conditions.

## 2. Results

### 2.1. Environmental Conditions

**Figure 1:**
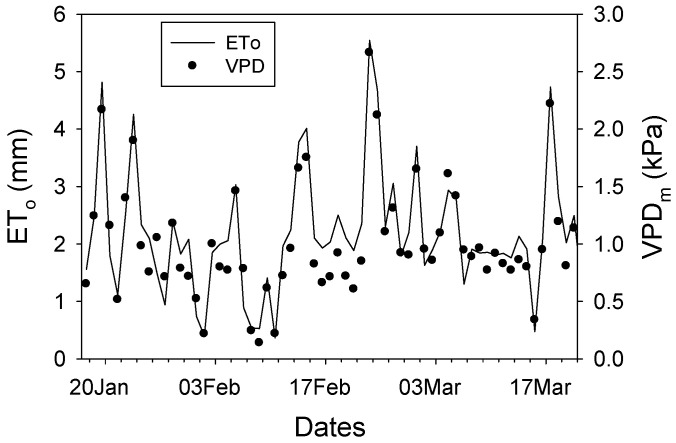
Eto (line) and midday VPD (circles) during the experimental period (17 January–23 March). Eto: evapotranspiration; VPD: vapor pressure deficit.

The experimental period showed variable environmental conditions, comprising sunny, cloudy, and rainy days. The average midday vapor pressure deficit (VPDm) was recorded at 0.99 kPa, with observed minimum and maximum values of 0.07 kPa on 8 February 2014, and 2.73 kPa on 22 February 2014, respectively (Figure 1). Additionally, reference evapotranspiration (Eto) demonstrated a similar trend, reaching its lowest values on 8 February and peaking on 22 February (Figure 1).

### 2.2. Water Relations

**Figure 2:**
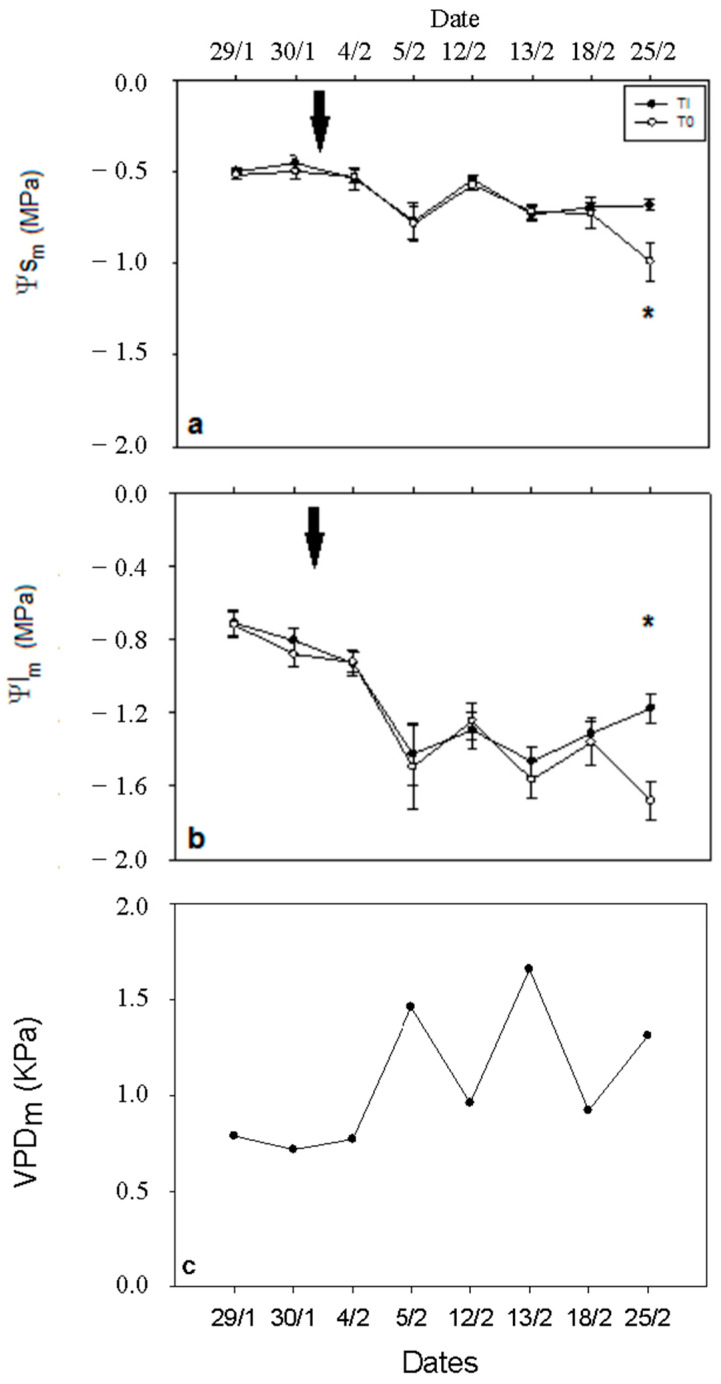
Stem (**a**) and leaf (**b**) water potential is being presented under full irrigation (TI; -•-) and non-irrigated treatment (T0; -o-). The black arrow indicates the day on which irrigation turned off for T0. Midday VPD is also shown for the above days (**c**). Asterisks (*) indicate a statistically significant difference between the two groups (n = 3, *p* < 0.05). Error bars represent SEM. ΨSm: midday stem water potential; Ψls: midday leaf water potential; VPDm: midday vapor pressure deficit.

Midday stem water potential (Ψs) was comparable across both treatments, with an average of −0.62 MPa, until 18 February 2014, when a declining trend was observed in the T0 treatment. By 25 February, Ψs for T0 trees significantly decreased to −1.03 MPa (Figure 2a). Similarly, midday leaf water potential (Ψl) demonstrated analogous variations, with an average of −1.14 MPa, consistently lower in the T0 treatment during the main experimental period, and reaching a minimum of −1.77 MPa on 25 February (Figure 2b). Additionally, both Ψs and Ψl were significantly correlated with VPD values throughout the experimental period (Figure 2c).

### 2.3. Transpiration and Stomatal Conductance

**Figure 3:**
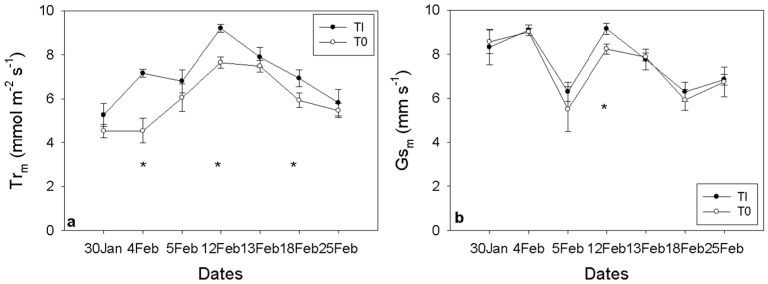
Midday leaf transpiration (Tr_m_) (**a**) and midday stomatal conductance (Gs_m_) (**b**) values at 7 different days (between 30 January and 25 February) for irrigation treatment (TI; -•-) and non-irrigated treatment (T0; -o-). Irrigation was turned off after the 31 January for T0. Asterisks indicate a statistically significant difference between the two groups (n = 3, *p* < 0.05). Error bars represent SEM.

Midday leaf transpiration was consistently higher in the TI treatment throughout the experimental period, with significant differences observed on 4, 12, and 18 February (Figure 3a). Midday stomatal conductance (Gs) was significantly greater for the TI treatment on 12 February; however, no significant differences were detected for the remaining days of the experimental season (Figure 3b).

### 2.4. SF and MDS Corelation

**Figure 4:**
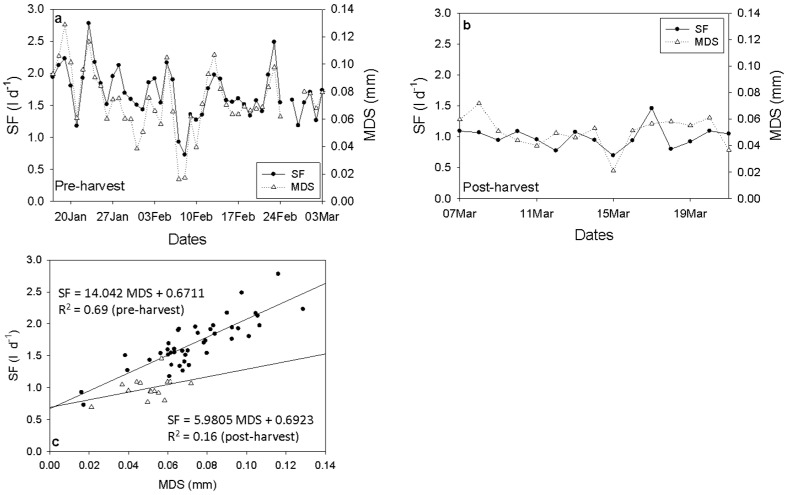
Trend of daily sap flow (SF) (-•-) and maximum daily shrinkage (MDS) (··Δ··) pre (**a**) and post-harvest (**b**) for TI. A linear regression between SF (•) and MDS (Δ) is also presented pre and post-harvest (**c**).

SF (l d^−1^) was highly correlated to MDS (mm) before harvest (r^2^ = 0.69) whereas it decreased substantially post-harvest (r^2^ = 0.16) (Figure 4).

### 2.5. Response of Branches to Mild Water Deficit

Pre harvest daily SF was normalized per unit of leaf area and fruit (DM (L m^−2^ kg^−1^ d^−1^). Post-harvest SF values were normalized per unit of leaf area (L m^−2^ d^−1^). Pre-harvest SF ranged between 0.89 and 3.42 L m^−2^ kg^−1^ d^−1^ for TI and between 0.41 and 2.92 L m^−2^ kg^−1^ d^−1^ for T0. Post-harvest SF values were between 0.95 and 1.92 L m^−2^ d^−1^ for TI and between 0.73 and 2.21 L m^−2^ d^−1^ for T0.

**Figure 5:**
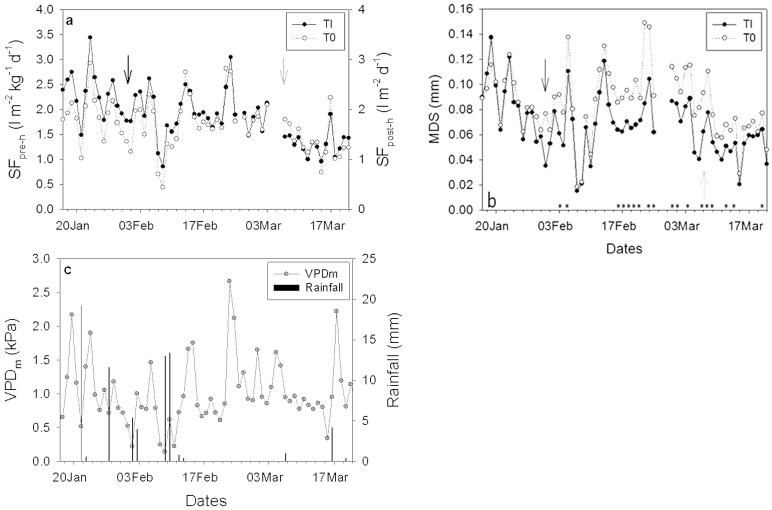
Daily sap flow (SF) pre-harvest (pre-h) (L m^−2^ kg^−1^ d^−1^) and post-harvest (post-h) (L m^−2^ d^−1^) (**a**) and maximum daily shrinkage (MDS) values for irrigation treatment (TI; -•-) and non-irrigated treatment (T0; ··o··) (**b**) during the experimental period (17 January–21 March). Rainfall (black bars) and midday vapor pressure deficit (VPDm; -•-) (**c**) values are also shown. The black arrow indicates the day when irrigation was turned off for T0 (31 January) while the gray arrow indicates the fruit harvest day (7 March). Asterisks (*) indicate a statistically significant difference between the two groups (n = 3, *p* < 0.05).

Lower SF values were monitored for T0 during the whole experimental period except DOY 43 (12 February) when midday stem water potential increased following precipitation of 37 mm (Figure 5). Both treatments resulted in had decreased SF values after fruit harvest (7–21 March) (Figure 5a). MDS values ranged from 0.02 to 0.14 for TI and from 0.02 to 0.16 for T0. MDS was higher in T0, showing having an increasing trend after DOY 31 (31 January) whereas decreased during DOY 36–43 (5–12 February) when rainfall occurred following an increase in water potential, while its values increased again, surpassing those of TI until the end of the experimental period (Figure 5b). Mild water deficit did not affect SF values but caused an increase in MDS with differences between treatments being significant for the main period after the DOY 31 and after the end of the rainfalls (Figure 5b,c). Both indicators were tightly correlated with midday VPD (). After the inhibition of irrigation for T0 and especially after the end of the rainfalls, MDS had higher values compared to TI and was more sensitive than SF. During post-harvest, both MDS and SF decreased. MDS maintained higher values in T0 (Figure 5b) while SF in T0 maintained lower values than TI (Figure 5a).

### 2.6. The Effect of Fruits on SF Rates and Branch Shrinkage (BS)

**Figure 6:**
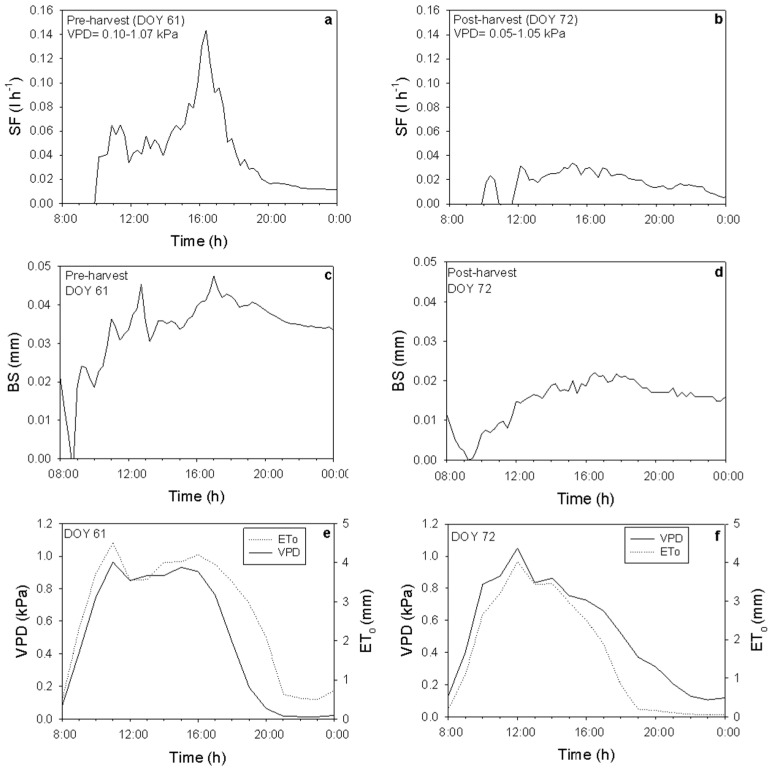
Daily sap flow (SF) and maximum daily shrinkage (MDS) values pre (**a**,**c**) and post-harvest (**b**,**d**) presented during two days with similar vapor pressure deficit (VPD) and evapotranspiration (ET_O_) values, one pre (**e**) and one post-harvest (**f**). Fruit harvest took place on day of year (DOY) 66 (31 January).

The impact of fruit on SF rates and BS was examined on two separate days with similar VPD and reference evapotranspiration (Eto), both before and after harvest (Figure 6e,f). SF rates were roughly 2.5 times higher prior to harvest, highlighting the significant effect of fruit on branch SF; after harvest, SF dropped by about 60% (Figure 6b). Conversely, BS values showed smaller variations during the post-harvest period, with a reduction of approximately 44% (Figure 6d).

### 2.7. SF-MDS and Climate Variables Correlation

**Figure 7:**
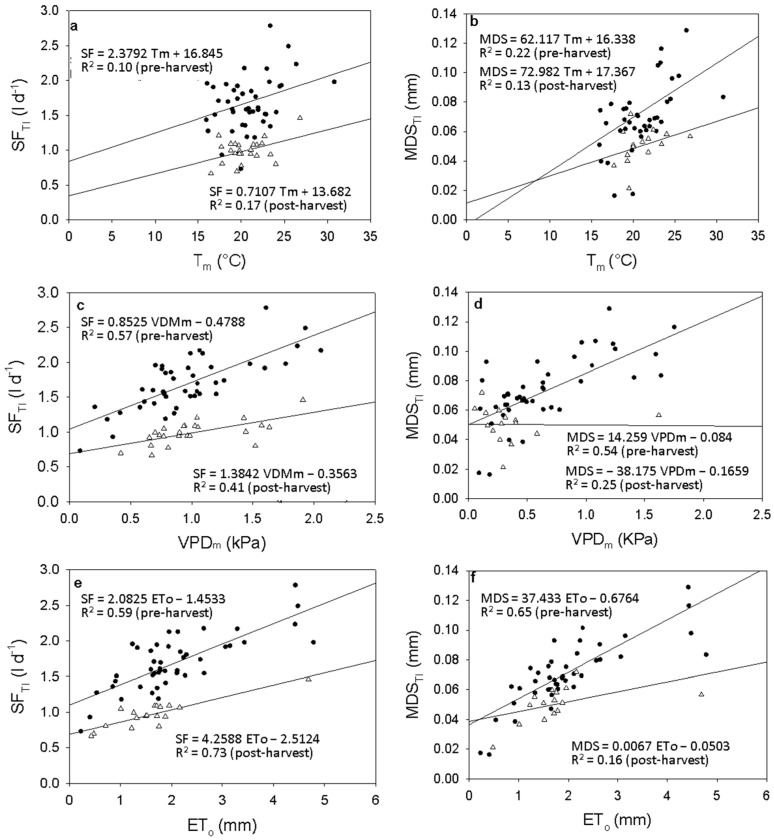
Relationships pre (•) and post (Δ)-harvest between daily sap flow (SF), midday air temperature T_m_ (**a**), midday vapor pressure deficit (VPD_m_) (**c**) and vapor pressure deficit (ET_o_) (**e**) during the experimental period and relationships pre (•) and post (Δ)-harvest between maximum daily trunk shrinkage (MDS) midday air temperature T_m_ (**b**) VPD_m_ (**d**) and Eto (**f**) for irrigated block (TI).

The SF and MDS measurements before and after harvest are shown in Figure 7, as a function of Tm, VPDm, and Eto. Pre-harvest increases in these environmental variables were linked to rises in SF and MDS. SF and MDS were strongly correlated with VPDm (r^2^ ~ 0.57 and 0.54) (Figure 7c,d) and Eto (r^2^ ~ 0.59 and 0.65) (Figure 7e,f) than with Tm (r^2^ ~ 0.10 and 0.22) (Figure 7a,b). After harvest, the regression between SF and Eto (r^2^ ~ 0.73) and Tm (r^2^ ~ 0.16) was higher than pre-harvest (Figure 7c), while it was lower between VPDm (r^2^ ~ 0.41) (Figure 7a,c). Regarding the relationship between MDS and climate variables post-harvest, it decreased significantly for all climate variables (Figure 7b,d,f).

### 2.8. SF and Water Status Indicators Correlation

On 18 February, SF was strongly correlated with leaf water potential (r^2^ ≈ 0.77), whereas its relationship with stem water potential was moderate (r^2^ ≈ 0.61) (Figure S1d,c). Relationships between SF and leaf transpiration (r^2^ ≈ 0.59) as well as stomatal conductance (r^2^ ≈ 0.43) were comparatively weaker (Figure S1a,b).

### 2.9. Irrigation, Soil Moisture and Rainfall

During the experimental period, the trees received a total of 1.07 m^3^ tree^−1^ of irrigation. Soil moisture was maintained at high levels, and irrigation volumes were based on evapotranspiration demands (Figure S2). Rainfall occurred on several days during the experimental period, reaching a total of 141.8 mm; the highest values were recorded on the 10 February (40.6 mm).

**Figure 8:**
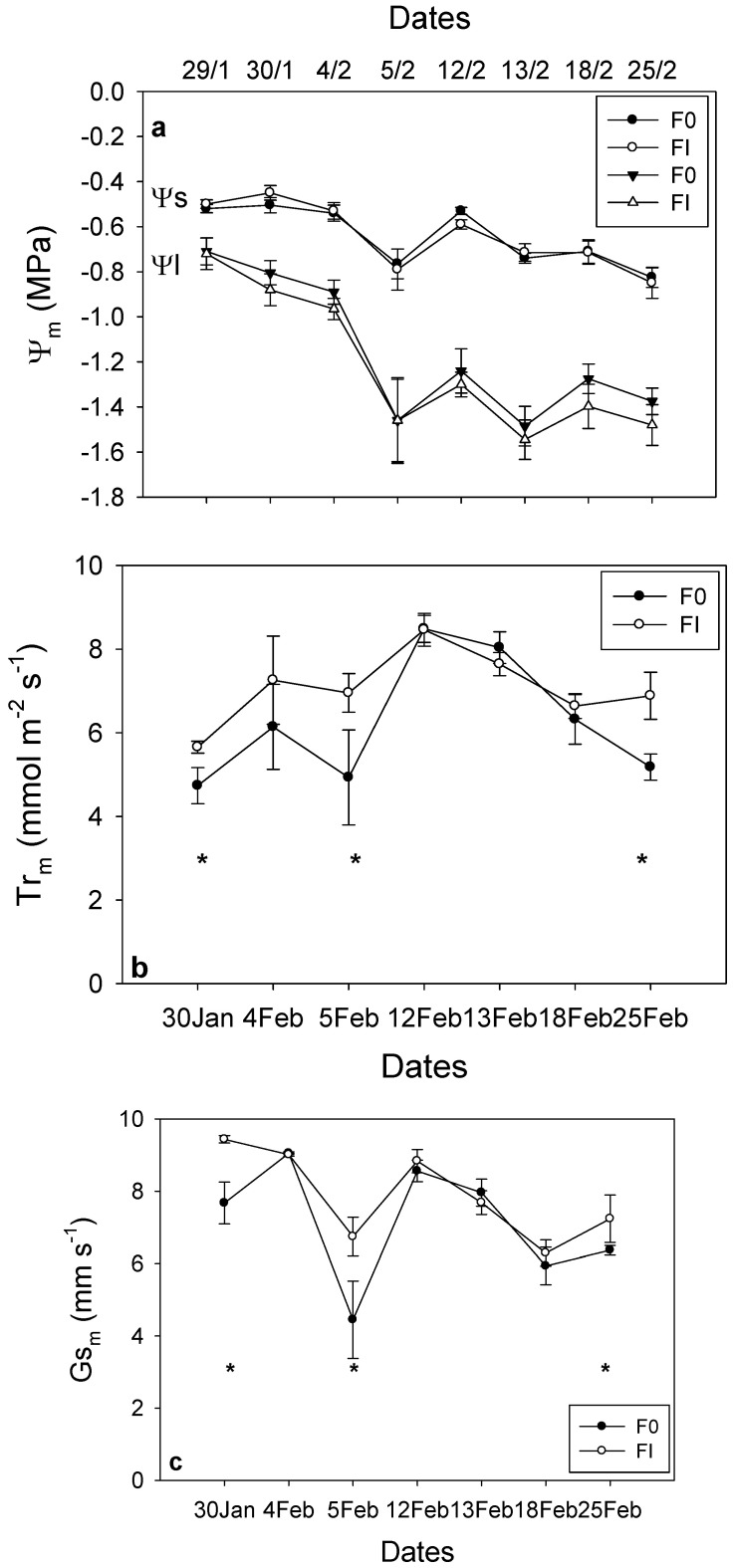
Midday stem (Ψs) water potential for F0 (-•-) and FI (-o-) and midday leaf (Ψl) water potential values for F0 (-▼-) and FI (-Δ-) during 8 different days from 29 January to 25 February (**a**). Moreover, values of midday leaf transpiration (Tr_m_) (**b**) and stomatal conductance (Gs_m_) (**c**) during 7 days between 30 January and 25 February are shown for F0 (-•-) and FI (-o-). Asterisks (*) indicate a statistically significant difference between the two groups (n = 3, *p* < 0.05). Error bars represent SEM.

Midday stem water potential (Ψs) values were similar for F0 and FI, whereas midday leaf water potential (Ψl) values were lower for FI during the main experimental period (Figure 8a). Midday leaf transpiration was higher for FI, with higher readings recorded on 30 January, 5 and 25 February (Figure 8b). Similarly, midday stomatal conductance (Gs) was higher for FI and values were significantly higher on 30 January and 5 February (Figure 8c).

**Figure 9:**
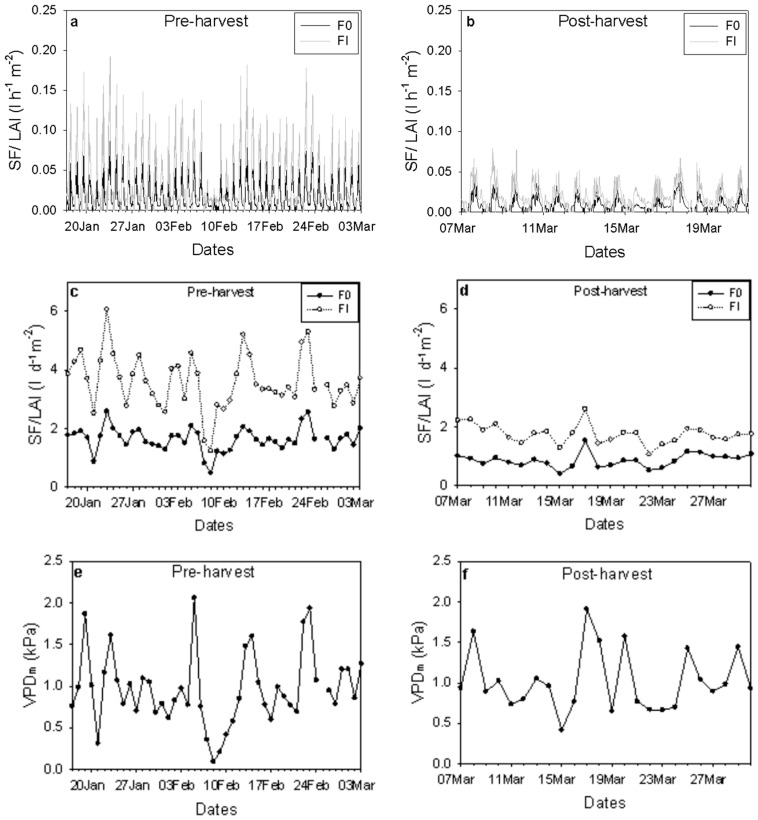
Values of hourly sap flow (SF) divided by leaf area index (LAI) pre and post-harvest (**a**,**b**) for F0 (black line) and FI (grey line) and daily SF divided by LAI pre and post-harvest (**c**,**d**) for F0 (-•-) and FI (-o-) are presented. Midday VPD values pre (**e**) and post-harvest (**f**) are also shown.

When SF was normalized by branch leaf area and fruit dry matter during the pre-harvest period, clear differences emerged between branch categories. Pre-harvest normalized SF (SF per unit LAI and fruit DM) was approximately 2.2-fold higher in FI branches compared to F0 branches (Figure 9a–d). After fruit harvest, SF was normalized by leaf area only, and FI branches maintained approximately two-fold higher SF than F0 branches (Figure 9b,d). Following harvest, SF declined markedly in both branch categories, decreasing to 52% of pre-harvest values in F0 and to 49% in FI (Figure 9b,d). In both pre- and post-harvest periods, normalized SF closely followed variations in midday VPD (Figure 9e,f).

### 2.10. The Effect of VPD on SF in Branches with Different LAI: Fruit DM Ratio

Three days of different VPD values were chosen in order to describe the response of SF to VPD in branches with different LAI: fruit DM ratio (Figure S3). The branches with lower LAI: fruit DM ratio (FI) maintained around 2.2-fold higher SF values during a rainy, cloudy and sunny day (Figure S3a–c). The SF was better correlated with VPD at higher values (sunny day) (Figure S3f). The FI had a better correlation with VPD during the rainy (r^2^ ~ 0.29) and cloudy day (r^2^ ~ 0.63) than F0 (r^2^ ~ 0.18 and 0.45) (Figure S3d,e), whereas the opposite was observed on happened during the sunny day (r^2^ = 082 for FI and r^2^ = 084 for F0) (Figure S3f).

## 3. Discussion

In this study, we aimed to elucidate the dynamics of SF and MDS in apple tree branches under varying fruit loads and water supply conditions. Our findings serve to enhance the scientific understanding of how these metrics are influenced by agricultural practices, particularly regarding irrigation management during critical developmental phases of apple cultivation. A notable objective was to assess the interrelationships between SF, MDS, environmental conditions, and physiological indicators of water status within the trees. By integrating branch-scale hydraulic measurements with physiological and climatic drivers, this work contributes to bridging the gap between mechanistic plant water relations and applied orchard water management.

Although the two treatments (TI and T0) exhibited similar SF rates before and immediately after irrigation withdrawal (Figure 5), this response reflects both the identical irrigation regime applied before DOY 31 and the mild, progressive nature of the induced water deficit. Under these conditions, SF remained largely governed by atmospheric demand and canopy transpiring area rather than soil water availability. Consequently, reductions in SF were not immediate following irrigation cessation, whereas MDS responded more rapidly and consistently. This divergence highlights that, at the branch scale and under mild water deficit, SF is less sensitive than stem diameter variation for detecting early changes in tree water status.

The pre-harvest results demonstrated a strong positive correlation between SF and MDS, which significantly diminished post-harvest. This phenomenon can be interpreted through the physiological principles governing tree water relations. The pronounced correlation observed prior to harvest suggests that the presence of fruits considerably augments the physiological demand for water within the tree. It has been proved that the increased water demand during fruit development is driven by several factors: the conversion of starches to sugars decreases osmotic potential, thereby drawing water into the fruit; the accumulation of sugars supports growth by maintaining turgor pressure; ongoing photosynthesis in leaves supplies carbohydrates, requiring a steady water supply; transpiration from fruits, similar to that from leaves, is influenced by environmental conditions; and the shift in resource allocation prioritizes water and nutrient transport to the fruit, potentially leading to water stress in other parts of the plant if water is limited [27,28,29,30]. Therefore, fruits increase transpiration rates, enhancing the flow of water through the tree’s vascular system [31], thereby establishing a greater reliance on SF as an indicator of tree hydration status.

Our findings is consistent with previous studies showing that fruit-bearing branches constitute strong hydraulic sinks, leading to increased xylem fluxes and enhanced use of internal water reserves. Increased fruit load has been reported to intensify diurnal sap flow and to amplify stem or branch diameter fluctuations as a result of greater depletion and subsequent nocturnal refilling of elastic tissues associated with fruit water demand [32,33]. Following fruit harvest, the reduced correlation between SF and MDS indicates a marked decline in physiological demand for water. This change is substantiated by the diminished fluctuations in both metrics, consistent with the physiological adjustments that occur in response to fruit removal. The post-harvest decoupling of SF and MDS highlights a shift from sink-driven to atmosphere-driven control of tree water use, as transpiration demand becomes dominated by leaf area and environmental conditions rather than reproductive sinks [34,35].

In addition to the relationship between SF and MDS, our findings underscore the impact of fruit load on SF rates and water consumption, as indicated by the contrasting LAI:fruit DM ratios. Branches characterized by lower LAI ratios displayed markedly higher SF rates, highlighting the significant role fruit load plays as a driver of water uptake and utilization in apple trees. This observation can be attributed to increased leaf transpiration coupled with lower midday leaf water potentials observed in these branches. Such patterns indicate a tighter coupling between carbon allocation and hydraulic function, where branches supporting higher fruit demand operate closer to hydraulic limits to sustain assimilate transport [36,37]. The physiological stress experienced by branches with lower LAI ratios suggests that they are working harder to maintain water balance, aligning with literature that indicates a direct correlation between fruit loading and increased water use rates in other fruit-bearing species [38]. This branch-scale heterogeneity emphasizes that whole-tree averages may obscure critical within-canopy differences relevant for precision irrigation strategies.

The investigation into midday leaf and stem water potentials revealed a substantially stronger correlation with SF compared to leaf transpiration and stomatal conductance. This finding underscores the notion that while stomatal functions and transpiration are critical components of water regulation, their responses are highly susceptible to acute environmental changes. In contrast, stem and leaf water potentials reflect more sustained water dynamics, which may offer insights into the overall health and hydration status of the tree. These results support the use of water potential measurements as integrative indicators of tree hydraulic status, particularly under fluctuating atmospheric demand [39]. The correlation between SF and midday environmental variables, such as VPD and ETo, further illustrates the intricate interplay between tree physiology and prevailing climatic conditions [40,41]. Notably, the tighter correlations observed during the pre-harvest phase may suggest that trees are better adapted to respond to these abiotic stressors when influenced by the presence of fruit. This enhanced sensitivity likely arises from increased hydraulic conductance and stomatal regulation aimed at sustaining fruit growth under variable climatic conditions.

The limited variation in midday stem water potential, despite pronounced fluctuations in soil moisture, warrants further consideration. The limited variation in midday stem water potential, despite pronounced fluctuations in soil moisture, warrants further consideration. Despite soil moisture occasionally exceeding field capacity or approaching permanent wilting point thresholds, midday stem water potential values remained within a relatively narrow range throughout the experimental period. This apparent decoupling likely reflects the large rooting volume and hydraulic capacitance of mature apple trees, which buffer short-term soil moisture fluctuations and delay the transmission of soil water deficits to aboveground tissues. Hydraulic capacitance, together with internal water storage in woody tissues, enables trees to temporarily maintain transpiration under declining soil water availability [42]. In such systems, plant water status can be strongly regulated by atmospheric demand (e.g., vapor pressure deficit) rather than by short-term changes in soil water availability. For example, in fruit orchards, midday stem water potential often correlates more closely with environmental demand than with soil water content, highlighting the influence of atmospheric conditions on water status measurements [43].

From a practical perspective, our results suggest that MDS serves as a more sensitive indicator of tree water status under mild water deficit conditions, particularly in branches characterized by lower LAI:fruit DM ratios. MDS responded earlier and more consistently than SF to irrigation withdrawal, indicating its suitability for detecting incipient water stress before reductions in transpiration become apparent, as previously reported by Ortuño et al. [44]. This insight is vital for refining water management strategies in apple orchards, as it provides growers with actionable data regarding optimal irrigation timing and quantification, necessary for sustaining tree health and ensuring fruit quality. By incorporating insights from MDS measurements alongside SF data, agricultural practices can be strategically modified to alleviate water stress during critical growth periods of fruit development. Such combined indicators may support the development of decision-support tools for precision irrigation, particularly under conditions of increasing climatic uncertainty.

This study is subject to several limitations that should be taken into account when interpreting the results. First, the number of instrumented trees was necessarily limited due to the intensive and invasive nature of branch-scale SF and MDS measurements. Consequently, the findings are best interpreted as providing mechanistic insights into branch-level hydraulic responses rather than broadly generalizable conclusions at the orchard scale. Second, although trees were managed under standard commercial practices and key environmental drivers during the experimental period were carefully monitored, long-term legacy effects of prior management and environmental exposure cannot be fully excluded in mature, field-grown trees. In addition, while the combined use of SF and MDS offers valuable insight into the interactions between fruit load, water supply, and tree water status, these indicators may not capture the full spectrum of physiological processes governing tree hydraulics across diverse environmental conditions, cultivars, or production systems. Future studies incorporating larger numbers of trees, multiple seasons, replicated orchard sites, and complementary measurements such as hydraulic vulnerability traits, non-structural carbohydrate dynamics, and long-term yield responses will be necessary to further resolve these mechanisms and to extend the applicability of these findings to a wider range of production contexts.

## 4. Materials and Methods

### 4.1. Experimental Site and Plant Material

The experiment was conducted on seven-year-old apple trees (*Malus domestica* Borkh.) cv. ‘Jazz™’ (registered cultivar name ‘Scifresh’), a commercial cultivar developed in New Zealand from a cross between ‘Royal Gala’ and ‘Braeburn’ and widely grown under licensed production systems. The cultivar is characterized by moderate-to-high vegetative vigor, a well-developed canopy, and a strong sink demand during fruit development, traits inherited from its parentage. ‘Jazz™’ produces medium-sized fruits with high firmness, elevated juice content, and a dense flesh structure, indicative of sustained water import and turgor maintenance during fruit growth. From a physiological perspective, the cultivar is known for active transpiration and carbohydrate allocation during the pre-harvest period, reflecting its high fruit dry matter accumulation. Agronomically, ‘Jazz™’ is widely cultivated under intensive orchard systems and exhibits good storability and yield stability across contrasting climatic regions, suggesting efficient water use and hydraulic regulation. These characteristics make ‘Jazz™’ a suitable model cultivar for investigating the interactions between fruit load, branch-scale SF, and stem diameter variations under contrasting water supply conditions.

The experiment took place over a period of 12 weeks during the summer of 2013–2014 in a commercial apple orchard (Mr Apple New Zealand Ltd.) located near Havelock North, New Zealand (39.59° S, 176.93° E). Orchard management followed commercial practices: inter-row areas were grassed, and a 1 m wide herbicide-treated strip was maintained beneath the tree rows.

Six seven-year-old apple trees were selected within the same orchard block. Trees were planted in north–south-oriented rows, with 4.5 m spacing between rows and 3.5 m spacing between trees within rows. Two adjacent rows were used for the study: one row was maintained under full irrigation (TI), while irrigation was completely withdrawn from the adjacent row (T0) after day of year (DOY) 31. Three trees per treatment were used, with each tree considered an independent experimental unit. Each tree was considered an experimental unit. Although irrigation treatments were imposed at the row level, trees were spatially separated and instrumented individually, and physiological measurements were conducted at the branch scale. In addition, continuous measurements taken over time provided repeated observations for each experimental unit, increasing the robustness of treatment comparisons.

One west-oriented branch per tree was selected for measurements, resulting in six monitored branches in total. Branch diameter was measured at the sensor installation point (approximately 10 cm from the branch base) and ranged from 1.5 to 2.2 cm. A schematic illustration and photographs of the orchard layout and sensor installation are provided in the Supplementary Materials (Figure S4). All monitored branches were subjected to the same irrigation regime; thus, branch categories did not differ in water supply or branch cross-sectional area. At the end of the experimental period, sap flow (SF) data were grouped according to the branch-level ratio of leaf area index (LAI) to fruit dry matter (DM). Based on this ratio, branches were classified as having either high LAI:fruit DM (0.79 m^2^ kg^−1^; F0, low fruit load) or low LAI:fruit DM (0.31 m^2^ kg^−1^; F1, high fruit load). This classification reflects differences in fruit load relative to leaf area and enabled assessment of fruit load effects at the branch level, thereby complementing the limited number of whole-tree replicates. Although controlled experiments using container-grown trees can minimize environmental variability, the use of mature, field-grown apple trees in this study allowed assessment of SF and stem diameter responses under realistic orchard conditions, thereby enhancing the relevance of the results for irrigation management in commercial production systems.

### 4.2. Irrigation and Soil Moisture Monitoring

Irrigation scheduling for the irrigated treatment (TI) was based on reference evapotranspiration (ETo) obtained from a nearby weather station and crop coefficients appropriate for mature apple orchards. Water was supplied using 27 L h^−1^ micro-jet sprinklers (Netafim Gyro Net; Tel Aviv, Israel), with one sprinkler installed for every two trees. Over the experimental period, the cumulative irrigation volume applied to TI trees was approximately 1.07 m^3^ tree^−1^.

For the non-irrigated treatment (T0), irrigation was completely suspended after day of year (DOY) 31 (31 January 2014) and remained withheld until the end of the experiment on 22 March 2014. During this period, trees relied exclusively on natural rainfall. The resulting irrigation contrast induced a mild and progressive soil water deficit rather than acute drought stress, as indicated by midday stem water potential values remaining above −1.2 MPa.

Soil volumetric water content was monitored throughout the experimental period using time-domain reflectometry (TDR) probes installed at multiple depths within the apple tree rooting zone. Sensors were distributed from the soil surface down to 1.1 m depth, and soil moisture data were averaged across the 0–90 cm profile, which represents the effective root zone considered for irrigation monitoring in this study. This depth-integrated soil moisture signal was used to evaluate soil water availability for the trees and to guide interpretation of the irrigation regime applied in the experiment. Measured values were interpreted relative to field water capacity (FWC) and permanent wilting point (PWP), previously determined using the pressure plate method [45], thereby providing a physiological context for plant water availability rather than relying on absolute soil moisture thresholds (Figure S5).

### 4.3. Fruit Dry Matter and LAI Determination

Total fruit fresh mass was determined on a per-tree basis. To quantify fruit dry matter content while capturing within-tree variability in fruit size, six fruits spanning the observed range of diameters within each tree were randomly selected from multiple canopy positions. Fruits were intentionally not sampled from the instrumented branches in order to avoid disturbing sap flow measurements and branch structure during the experimental period. The selected fruits encompassed the natural size variability within a tree and were oven-dried at 105 °C for 36 h to determine dry matter content, which was subsequently used to estimate total fruit dry matter at the tree level.

The leaf area index of the branches was measured at the end of the experiment by sampling 1/50 of the leaves. Afterwards, the leaves were transferred to the lab and analyzed by a Licor belt-feed leaf area meter (Model 3100; Licor Ltd., Lincoln, NE, USA), multiplying the result by 50.

### 4.4. Leaf Gas Exchange, Water Potential, and Climate

Leaf gas exchange was assessed using an AP4 porometer, which measures stomatal conductance to water vapor. Under moderate relative humidity conditions (40–70%), transpiration rates were estimated from stomatal conductance following standard diffusion equations [46]. Measurements were avoided under extreme humidity to minimize uncertainty in transpiration estimates.

Leaf and stem water potentials were measured concurrently with gas exchange assessments using a Scholander pressure chamber [47]. For stem water potential, leaves were shaded with aluminum foil for approximately one hour prior to measurement. Climate data were sourced from a local weather station near the experimental site.

### 4.5. Sap Flow Measurement

SF was measured using the CHPM as folly described by Madurapperuma et al. [48]. This method involved a heater probe and two asymmetrical temperature sensors installed on the trunk tissue, positioned 5 mm upstream and 10 mm downstream from the heater. Probes were radially mounted at the base of branches near the central trunk, aligned parallel to the SF direction. The heater probes were constructed from 18-gauge stainless steel rods, measuring 20 cm in length and 1.3 mm in diameter, with a Teflon insulation tube containing a 13 ohm/m nichrome resistance wire. The temperature sensors were approximately 2 mm in length and 0.3 mm in diameter. All sensors and probes were connected to a CR1000 data logger (Campbell Scientific, Logan, UT, USA), which delivered a 2-s heat pulse to the probes every 15 min. The data logger calculated the raw heat pulse velocity by recording the crossover time (t_z_, s), defined as the time required for the temperature increases at the upstream and downstream sensors to become equal. Heat pulse velocity (V_z_, cm s^−1^) was calculated as shown in Equation (1)

(1) $$Vz\;=\left(\frac{Xd+Xu}{2tz}\right)$$

where X_d_ and X_u_ (cm) are the distances between the heater and the upstream and downstream temperature probes, respectively. This formulation assumes symmetrical heat transport around the heater probe and is valid under steady-state sap flow conditions. Values of t_z_ data were collected weekly, and subsequent corrections were applied to account for probe-induced wounding and thermal diffusivity effects [49,50].

### 4.6. Wound Corrections

The installation of probes may cause mechanical damage to xylem tissues, leading to non-conductive zones that affect SF rates [21]. To correct raw heat pulse velocity (V_z_) for wound effects, a corrected heat velocity (V_c_) was calculated using the empirical correction proposed by Swanson and Whitfield [50]:

(2) Vc=a+bV+cV

where *a*, *b*, and *c* are empirically derived coefficients that depend on wound diameter and probe geometry. This correction accounts for altered heat transport pathways caused by probe installation and is essential for obtaining physiologically meaningful SF estimates, particularly at low flow rates.

### 4.7. Branch Diameter Variations

Micrometric fluctuations in branch diameter were monitored concurrently with SF measurements using linear variable displacement transducers (LVDTs; Model DF ± 2.5 mm, accuracy ± 10 μm; Solartron Metrology, Bognor Regis, UK). The LVDTs, constructed from Invar (low-thermal-expansion alloy) and aluminum, were secured to the branches using adjustable brackets and shielded with reflective foil to minimize thermal artifacts. Measurements were recorded every 2 s and averaged over 15 min intervals using a CR10X data logger equipped with an AM25T multiplexer (Campbell Scientific).

Daily diameter fluctuation indices included maximum daily trunk diameter (MXTD), minimum daily trunk diameter (MNTD), and maximum daily shrinkage (MDS), calculated as follows:

(3) MDS = MXTD − MNTD

As described by Han et al. [51]. MDS was used as an indicator of tree water status, reflecting the balance between xylem tension during transpiration and nocturnal tissue rehydration.

### 4.8. Conversion of Heat-Pulse Velocity to SF Density

Following the correction of *V*_c_, actual SF can be calculated. According to Marshall [52] when sap and wood are theoretically adjusted to these of an undisturbed plant, sap flux density, *J* (m s^−1^), can be calculated from:

(4) $$J=P\;\left(0.33+M\right)Vc$$

where *P* (kg m^−3^) is the density of the wood (dry weight of wood/green volume) and *M* is the sapwood moisture content (kg kg^−1^). The constant 0.33 represents the specific heat of dry wood relative to water.

Alternatively, as described by Edwards and Warwick [53], SF density can be expressed by explicitly considering the three phases of sapwood (solid, liquid, and gas) as shown in Equation (5):

(5) $$J=(0.505\;F_{M}\;+F_{L})\;V_{c}$$

where *F_L_* and *F_M_* are the volumetric fractions of liquid (sap) and solid (wood) phases, respectively, and 0.505 is a species-independent coefficient describing sapwood’s thermal properties.

### 4.9. Estimating Volumetric SF

The sap flux density values derived from Equation (5) represent point measurements within the sapwood. Because sap flow is radially heterogeneous, typically peaking within the outer sapwood (10–20 mm from the cambium), measurements at multiple depths are required for accurate scaling [54].

Volumetric SF was then obtained by integrating J(γ) over the conductive sapwood area:

(6) J(γ) = α + βγ + δγ^2^

where γ (m) is the radial distance from the stem center, and α, β, δ are regression coefficients obtained from least-squares fitting.

To estimate whole-branch volumetric SF (Q, m^3^ s^−1^), SF density was expressed as a function of stem radius (γ, m) using a least-squares regression:

(7) $$Q=2\pi \;\int \int_{H}^{R}\;J\left(\gamma \right)\gamma \;d\gamma$$

where R (m) is the cambium radius defining the outer boundary of the sapwood, H (m) is the heartwood radius defining the inner boundary of the conductive xylem, and γ (m) represents the radial distance from the stem center. Heartwood radius was determined from branch core samples, while cambium radius was estimated from stem circumference after correcting for bark thickness. This integration approach follows the weighted-average method described by Hatton et al. [55] and allows scaling of point sap flux density measurements to whole-branch volumetric SF.

### 4.10. Statistical Analysis

Statistical analyses were conducted using Minitab 16. Differences between irrigation treatments (TI vs. T0) and between branch categories (F0 vs. FI) were assessed using two-sample *t*-tests. Before analysis, data normality and homoscedasticity were verified using Shapiro–Wilk and Levene’s tests, respectively. Relationships between physiological variables and environmental drivers were evaluated using linear regression analysis, with coefficients of determination (r^2^) reported. Significance was accepted at *p* < 0.05.

## 5. Conclusions

In summary, this study indicates that branch-scale hydraulic indicators provide complementary information for irrigation management in mature apple orchards. Under mild and progressive soil water deficits, SF remained largely governed by atmospheric demand and fruit load, whereas MDS responded earlier and more consistently to irrigation withdrawal. These results support the use of MDS as an early-warning indicator for scheduling irrigation when incipient water stress develops, while SF measurements are better suited for quantifying transpiration dynamics and fruit load effects. Future studies including larger tree numbers and multiple seasons are needed to refine quantitative irrigation thresholds and to scale these findings to orchard-wide irrigation strategies.

## Data Availability

The datasets generated during and analyzed during the current study are available from the corresponding author on reasonable request.

## Supplementary Materials

The following supporting information can be downloaded at https://www.mdpi.com/article/10.3390/plants15040608/s1, Figure S1: Relationships between hourly SF measured in the branches of the irrigated block (TI) and water status indicators (Leaf transpiration (**a**), Stomatal conductance (**b**), Stem (**c**) and leaf (**d**) water potential) on 18 February 2014; Figure S2: Values of irrigation (black bars) and soil moisture at 0–110 cm depth (gray line) during the experimental period; Figure S3: Hourly values of SF/LAI for F0 (black line) and FI (grey line), between 8am to midnight during a rainy (**a**), cloudy (**b**) and a sunny day (**c**). Relationships between VPD versus FI (·) and versus F0 (o) are also shown for a rainy (**d**), cloudy (**e**) and a sunny day (**f**); Figure S4: Experimental setup, orchard conditions, and sensor placement used in this study. (**a**) Schematic illustration of branch-scale sap flow measurements, showing the position of the sap flow gauge installed near the base of a three-year-old fruit-bearing branch. (**b**) Photograph of the commercial apple orchard (Malus domestica Borkh. cv. ‘Jazz™’) illustrating tree spacing, row orientation, and field-grown conditions under which the experiment was conducted. (**c**) Sap flow sensor components before installation, including probes and needles, shown with a scale ruler to indicate their dimensions. (**d**) Example of a heat-balance sap flow gauge installed on a west-oriented branch, approximately 10 cm from the branch base. (**e**) Close-up view of a fruit-bearing branch equipped with stem diameter variation (dendrometer) sensors, illustrating simultaneous measurements of branch hydraulic activity and fruit load; Figure S5: Permanent wilting point (PWP) and field water capacity (FWC).
