# Supplementary material for: Sap Flow Variability in Malus domestica Borkh. (‘JazzTM’) Trees Under Differing Water Supply Conditions and Fruit Loads
Source: Plants (Basel). 2026 Feb 14;15(4):608. doi: 10.3390/plants15040608 (PMC12944478; doi:10.3390/plants15040608)
Supplement: Supplementary file 1 [file plants-15-00608-s001.zip › plants-4097993-supplementary.pdf]

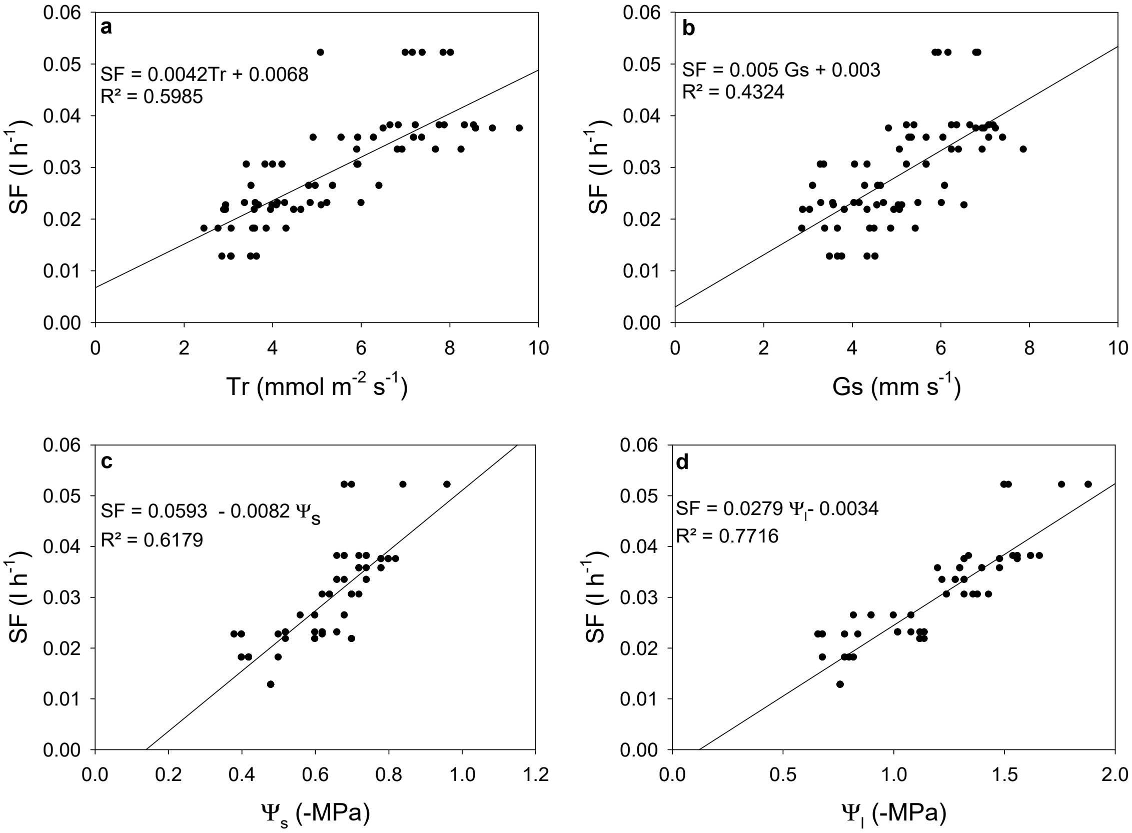

Figure S1. Relationships between hourly SF measured in the branches of the irrigated block (TI) and water status indicators (Leaf transpiration (a), Stomatal conductance (b), Stem (c) and leaf (d) water potential) on 18 February 2014.

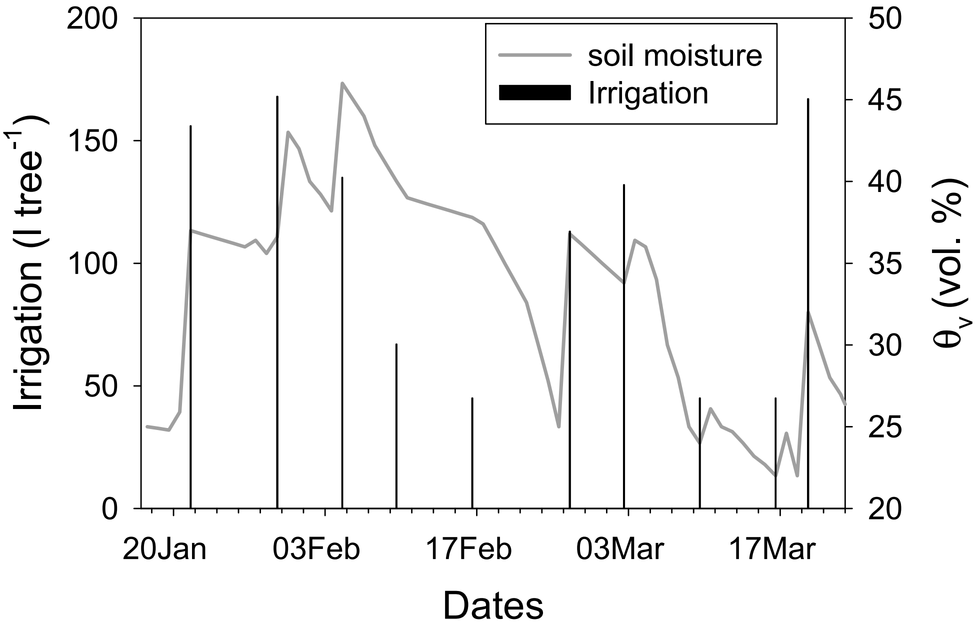

Figure S2. Values of irrigation (black bars) and soil moisture at 0-110 cm depth (gray line) during the experimental period.

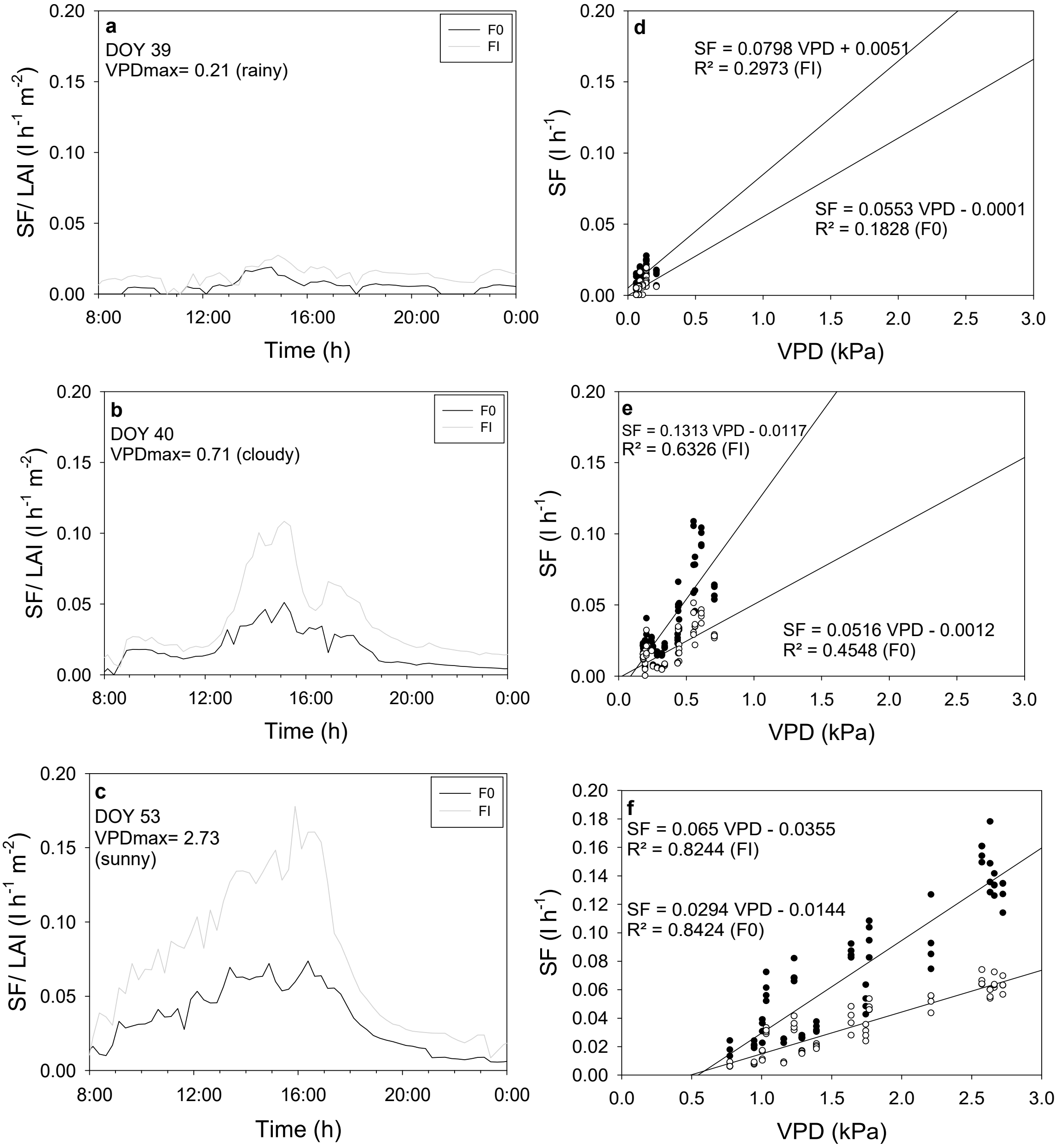

Figure S3. Hourly values of SF/LAI for F0 (black line) and FI (grey line), between 8am to midnight during a rainy (a), cloudy (b) and a sunny day (c). Relationships between VPD versus FI (•) and versus F0 (o) are also shown for a rainy (d), cloudy (e) and a sunny day (f).

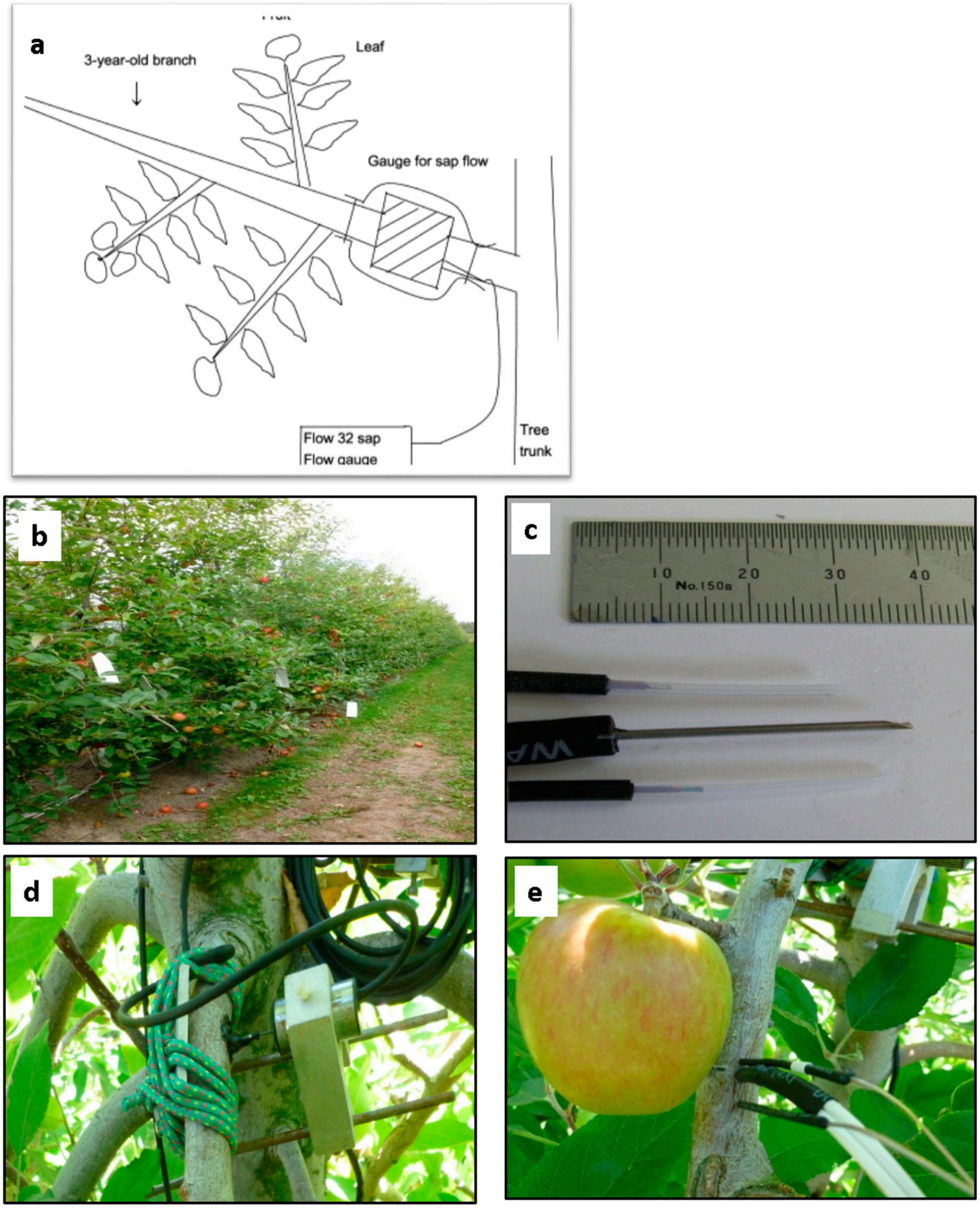

Figure S4. Experimental setup, orchard conditions, and sensor placement used in this study. (a) Schematic illustration of branch-scale sap flow measurements, showing the position of the sap flow gauge installed near the base of a three-year-old fruit-bearing branch. (b) Photograph of the commercial apple orchard (Malus domestica Borkh. cv. 'Jazz™') illustrating tree spacing, row orientation, and field-grown conditions under which the experiment was conducted. (c) Sap flow sensor components before installation, including probes and needles, shown with a scale ruler to indicate their dimensions. (d) Example of a heat-balance sap flow gauge installed on a west-oriented branch, approximately 10 cm from the branch base. (e) Close-up view of a fruit-bearing

branch equipped with stem diameter variation (dendrometer) sensors, illustrating simultaneous measurements of branch hydraulic activity and fruit load.

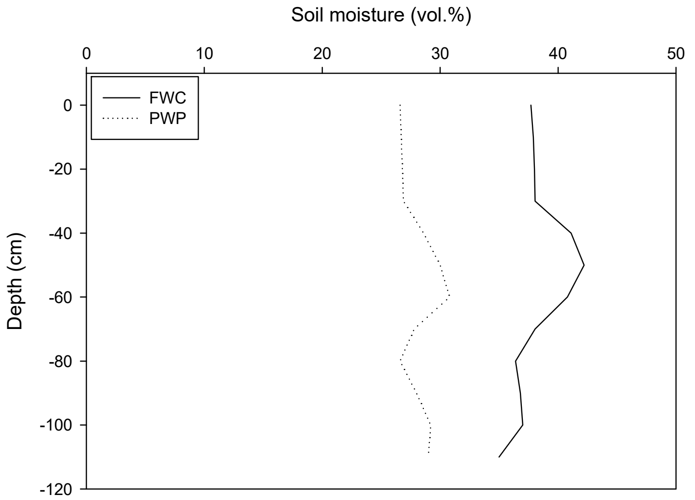

Figure S5. Permanent wilting point (PWP) and field water capacity (FWC)
